# *Brucella* NpeA is a secreted Type IV effector containing an N-WASP-binding short linear motif that promotes niche formation

**DOI:** 10.1128/mbio.00726-24

**Published:** 2024-06-07

**Authors:** Agostina Giménez, Mariela G. Del Giudice, Paula V. López, Francisco Guaimas, Hugo Sámano-Sánchez, Toby J. Gibson, Lucía B. Chemes, Carlos O. Arregui, Juan E. Ugalde, Cecilia Czibener

**Affiliations:** 1 Instituto de Investigaciones Biotecnológicas, Universidad Nacional de San Martín (UNSAM)-Consejo Nacional de Investigaciones Científicas y Técnicas (CONICET), Escuela de Bio y Nanotecnologías (EByN), Universidad Nacional de San Martín, San Martín, Buenos Aires, Argentina; 2 Zhejiang University School of Medicine, International Campus, Zhejiang University, Haining, China; 3 Biomedical Sciences, Edinburgh Medical School, The University of Edinburgh, Edinburgh, United Kingdom; 4 Structural and Computational Biology Unit, European Molecular Biology Laboratory, Heidelberg, Germany; Yale University School of Medicine, New Haven, Connecticut, USA

**Keywords:** *Brucella*, Type IV effector, N-WASP, *bab2_0195 *gene

## Abstract

The modulation of actin polymerization is a common theme among microbial pathogens. Even though microorganisms show a wide repertoire of strategies to subvert the activity of actin, most of them converge in the ones that activate nucleating factors, such as the Arp2/3 complex. *Brucella* spp. are intracellular pathogens capable of establishing chronic infections in their hosts. The ability to subvert the host cell response is dependent on the capacity of the bacterium to attach, invade, avoid degradation in the phagocytic compartment, replicate in an endoplasmic reticulum-derived compartment and egress. Even though a significant number of mechanisms deployed by *Brucella* in these different phases have been identified and characterized, none of them have been described to target actin as a cellular component. In this manuscript, we describe the identification of a novel virulence factor (NpeA) that promotes niche formation. NpeA harbors a short linear motif (SLiM) present within an amphipathic alpha helix that has been described to bind the GTPase-binding domain (GBD) of N-WASP and stabilizes the autoinhibited state. Our results show that NpeA is secreted in a Type IV secretion system-dependent manner and that deletion of the gene diminishes the intracellular replication capacity of the bacterium. *In vitro* and *ex vivo* experiments demonstrate that NpeA binds N-WASP and that the short linear motif is required for the biological activity of the protein.

**IMPORTANCE:**

The modulation of actin-binding effectors that regulate the activity of this fundamental cellular protein is a common theme among bacterial pathogens. The neural Wiskott–Aldrich syndrome protein (N-WASP) is a protein that several pathogens target to hijack actin dynamics. The highly adapted intracellular bacterium *Brucella* has evolved a wide repertoire of virulence factors that modulate many activities of the host cell to establish successful intracellular replication niches, but, to date, no effector proteins have been implicated in the modulation of actin dynamics. We present here the identification of a virulence factor that harbors a short linear motif (SLiM) present within an amphipathic alpha helix that has been described to bind the GTPase-binding domain (GBD) of N-WASP stabilizing its autoinhibited state. We demonstrate that this protein is a Type IV secretion effector that targets N-WASP-promoting intracellular survival and niche formation.

## INTRODUCTION

Bacterial pathogens are extremely versatile microorganisms that have evolved a plethora of virulence strategies to subvert host cell functions and establish successful infections. Due to the many different lifestyles these pathogens display, the mechanisms they use and the cellular pathways they exploit are very diverse (1). In the particular case of intracellular microorganisms, they must adhere, invade, survive the lytic environment, replicate in a permissive structure, and, finally, egress to reinitiate the infective cycle. Each stage of the intracellular cycle involves a battery of virulence proteins that modulate different host-cell activities. A common theme in many bacterial pathogens is their capacity to alter and/or modulate the actin cytoskeleton at different levels (2, 3). The assembly of specific actin arrays is essential for many fundamental cellular processes, including organelle organization (4), traffic and membrane dynamics (5, 6), and motility (7). These actin arrays are controlled by a complex network of actin-binding proteins which regulate the polymeric state, the nucleation and assembly of new filaments, the elongation and capping at the ends, the fragmentation and crosslink of filaments, and their anchoring to membranes (8). Intracellular bacterial pathogens have evolved a variety of mechanisms to modulate actin assembly, but they frequently converge in those that activate actin-nucleating factors, such as the actin-related protein Arp2/3 complex (9, 10). The Arp2/3 complex is intrinsically inactive, and a variety of nucleation-promoting factors (NPFs) can activate it (2). The neural Wiskott–Aldrich syndrome protein (N-WASP) is a NPF expressed ubiquitously, and a potent activator of Arp2/3 (11, 12). N-WASP has a modular domain organization consisting of an N-terminal WASP homology (WH1) domain, a GTPase-binding domain (GBD), a proline-rich domain, and a VCA (for Verprolin-homology, Central, Acidic) domain involved in binding and activation of Arp2/3 (13–15). Under basal conditions, the recruitment of actin to the Arp2/3 complex is prevented by an allosteric autoinhibition of N-WASP, mediated by an intramolecular interaction between the GBD domain and the α5 helix of the VCA domain (16). The auto-inhibited state is released by the upstream activator Cdc42-GTP, which interacts with the GBD domain and displaces the α5 helix of the C region, leaving the CA-terminal of the VCA domain accessible to the Arp2/3 complex (16, 17).

Several pathogens express virulence factors mimicking short linear motifs (SLiMs) in host cell proteins (18, 19). SLiMs are short stretches of amino acids often present in disordered regions of proteins which play important roles in cell signaling (20). SLiMs interact with their binding partners with moderate affinity, in the low micromolar range, and contribute to distinct functional conformations of a protein by stabilizing intramolecular interactions (21). One such SLiM, which can be represented by the regular expression [ILV][VA][^P][^P][LI][^P][^P][^P][LM], is known as the GBD C-helix motif and is found in the amphipathic α5 helix of the WASP and N-WASP VCA domain (15, 16). The GBD C-helix motif mediates N-WASP autoinhibition by binding internally to the N-WASP GBD domain. The GBD C-helix motif is mimicked by many virulence factors including the EspFu protein of enteropathogenic *Escherichia coli* (22–24), the IcsA factor of *Shigella flexneri* (25), and the TmeA protein of *Chlamydia trachomatis* (26). A common feature of all these factors is the capacity to activate N-WASP by intermolecular competition with the autoinhibitory interaction involving the GBD domain, which results in Arp2/3-dependent actin polymerization.

*Brucella* is a gram-negative facultative intracellular pathogen that causes brucellosis, a widespread zoonosis of animal and human health concern (27, 28). The hallmark of the infectious process of *Brucella* is its capacity to invade, survive, and replicate in professional and non-professional phagocytes (29, 30), activities that are many times dependent on effector proteins secreted or translocated by the Type IV (VirB) secretion system (T4SS) (27, 29). In the past decades, although many effector proteins have been identified in *Brucella* (29–32), the functions or molecular targets for most of them remain unknown. Particularly, to date, no virulence factors involved in actin modulation have been identified in *Brucella*.

In the present manuscript, we describe the identification of NpeA, a novel *Brucella* Type IV secreted effector with a SLiM predicted to mimic the GBD C-helix motif present in N-WASP. We show that this protein is secreted in a Type IV-VirB secretion-dependent manner that a null mutation of its coding gene affects *Brucella* replication in host-cells and that its overexpression facilitates intracellular replication. We further demonstrate that this novel effector binds N-WASP and propose that it functions by promoting the formation of replicative niches.

## MATERIALS AND METHODS

### Media and culture condition

*Brucella* strains were grown at 37°C in tryptic soy broth (TSB). *E. coli* strains were grown at 37°C in Luria–Bertani broth. If necessary, media were supplemented with the appropriate antibiotics at the indicated final concentrations: ampicillin, 100 µg/mL; kanamycin, 50 µg/mL; and nalidixic acid, 5 µg/mL.

### Intracellular replication assays

Antibiotic protection assays were performed in the HeLa cell line as described in reference 33. Cells were seeded in 24-well plates in suitable culture medium at 10^5^ cells/mL and incubated overnight at 37°C. *Brucella* strains were grown for 24 h and diluted in culture medium prior to infection. The suspension was added at a multiplicity of infection of 1,000:1 and centrifuged at 300 × *g* for 10 min. After 1 h of incubation at 37°C, cells were washed and fresh medium containing 100 µg/mL of streptomycin and 50 µg/mL of gentamicin was added. At 4, 24, and 48 h post-infection, cells were washed and lysed with 0.1% Triton-100X. The intracellular CFUs were determined by direct plating on TSB agar plates.

### Anti N-WASP antibody production

Anti-N-WASP antiserum was generated inoculating mice with the recombinant protein MBP-N-WASP with one primo inoculation (50 µg) in incomplete Freund’s adjuvant and two boosters (at 21 and 42 days) with 10 µg of the recombinant protein in incomplete Freund‘s adjuvant. Fifteen days after the second inoculation, the animals were euthanized, and the serum extracted and evaluated by Western blot. Mouse immunizations were approved by the local regulatory agencies (CICUAE-UNSAM).

### Plasmids and mutant construction

#### Construction of the *B. abortus* Δ*bab2_0195* mutant strain

Regions flanking the *bab2_0195* gene were amplified and ligated using the recombinant PCR technique (34). The resulting fragment was digested with BamHI and XbaI and ligated to the pK18mobSacB plasmid digested with the same enzymes. The primers used for PCR amplification were 5′-CGCGGATCCTCGTCACTGGAAGCAGCA-3′ and 5′-TAGCGCCACTTCTTTTACGC-3′ to amplify a 500 bp upstream region and 5′-AAAGAAGTGGCGCTATAGAGCATCCGGGACGGAAGT-3′ and 5′-GCTCTAGACCAGAAGAAGCTATAGATTG-3′ to amplify a 500 bp downstream region. The resulting plasmid was introduced in *B. abortus* by conjugation and the simple recombination event as well as the excision event selected as we have previously described. The deletion mutant was confirmed by colony PCR.

#### Construction of plasmid pBBR4-MCS4-Bab2_0195-3xFlag

To construct plasmid pBBR1-MCS4-Bab2_0195-3xFlag primers 5′-CTAGCTAGCAGTTGCCGGTTTCTTCCG-3′ and 5´-CGGAATTCGTTCCACGGGAGATAATC-3´ were used to amplify the *bab2_0195* gene from genomic DNA and cloned in the pBBR1-MCS4-3xFlag (35) in the NheI and EcoRI sites.

#### Construction of plasmid pBBR1-MCS4-NpeA_ΔSLiM_

To construct plasmid pBBR1-MCS4-NpeA_ΔSLiM_ expressing a NpeA version lacking the short linear motif sequence VADNIRTAL, primers 5′-AGTTGCCGGTTTCTTCCGGCAAGGCTGGCTAAAACAGTTTTGGCGAAGAG AAATTAGCAGGCGTAAAAGAAGTGGCGCTA-3′ and 5′-agcatcctggagcactggtg-3′ were used to amplify the region upstream of the motif and 5′-gtgctccaggatgctccgcgcggcaacagccgccgt-3′ and 5′-CGGAATTCGTTCCACGGGAGATAATC-3′ to amplify the region downstream. The two fragments were ligated using the recombinant PCR technique. The resulting fragment (complete NpeA lacking the SLiM sequence) was digested with NheI and EcoRI and cloned in the pBBR1-MCS4-3xFlag vector.

#### Construction of the plasmid for MBP-N-WASP expression

N-WASP was amplified by PCR from plasmid pYFP-C1-N-WASP (36) with primers 5′-CCGGAATTCATGAGCTCCGTCCAGCAG-3′ and 5′-GGAAGCTTTCAGTCTTCCCACTCATCATC-3′ and the resulting fragment cloned in the vector pMALC2x (New England Biolabs) in the EcoRI and HindIII restriction sites.

#### Construction of the plasmid for NpeA(90-70)-6×His expression

The NpeA DNA fragment coding a region from amino acids 90–170 was synthesized (Genscript) and cloned in the vector pET32b (Novagen) in the BamHI and EcoRI sites.

### Protein expression and purification

Recombinant poly-histidine-tagged NpeA(90-170)-6×His protein was expressed in *E. coli* and purified using nickel-affinity chromatography. Briefly, *E. coli* BL21 (DE3)pLYSs was grown at 37°C and expression induced with 1 mM IPTG (isopropyl-β-d-thiogalactopyranoside) at OD_600_ of 0.6. 3 h post-induction, cells were harvested, lysed by sonication, and the clarified extract injected in a nickel affinity chromatography column. After extensive washings, the protein was eluted with 150 mM imidazole and the eluates dialyzed against PBS to eliminate the imidazole.

Recombinant MBP-N-WASP protein was expressed in *E. coli* and purified using a maltose affinity column. *E. coli* BL21 (DE3)pLYSs was grown at 37°C and expression induced with 1 mM IPTG (isopropyl-β-d-thiogalactopyranoside) at OD_600_ of 0.6. 3 h post-induction, cells were harvested, lysed by sonication, and the clarified extract injected in a maltose affinity column. After extensive washings, the protein was eluted with 10 mM maltose and the eluates dialyzed against PBS to eliminate the maltose.

### Microscopy procedures

#### Secretion of NpeA

HeLa cells were infected with the strains expressing NpeA-3×Flag (multiplicity of infection [MOI], 1,000:1). At 2 h post infection, cells were washed three times with PBS and fixed for 15 min in 4% paraformaldehyde and processed for immunofluorescence labeling using rabbit α-*Brucella* polyclonal antibody (dilution, 1:1,500) and α-Flag monoclonal M2 antibody (Sigma, dilution, 1:4,000). The secondary antibodies used were goat anti-mouse Alexa Fluor 488 and goat anti-rabbit Alexa Fluor 568 (Molecular Probes, Invitrogen Co.) at a 1:4,000 dilution. For DNA staining, DAPI dye at 0.5 mg/mL (final concentration) was used. Co-localization was determined by quantifying the number of bacteria positive for the Flag staining.

#### Niche quantification

To determine the number of niches, HeLa cells were infected as described above (intracellular replication assays), and at 48 h post-infection, the cells were fixed for 15 min in 4% paraformaldehyde and processed for immunofluorescence labeling using rabbit α-*Brucella* polyclonal antibody (dilution, 1:1,500) and phalloidin. The secondary antibody used was goat anti-rabbit Alexa Fluor 568 (Molecular Probes, Invitrogen Co.) at a 1:4,000 dilution. For quantification purposes a group of 5 or more bacteria was considered a niche.

#### N-WASP association

The association of host N-WASP with different *Brucella* strains was analyzed in infected HeLa cells by confocal microscopy. At the end of the infection time, the preparation was washed with PBS (137 mM NaCl, 2.7 mM KCl, 10 mM Na_2_HPO_4_, and 1.8 mM KH_2_PO_4_ [pH 7.4]) and fixed with 4% paraformaldehyde in PBS for 20 min. Then, cells were permeabilized with 0.5% Triton X-100/PBS for 5 min and blocked in 3% bovine serum albumin (BSA/PBS) for 1 h. Primary and secondary antibodies (anti-*Brucella* polyclonal rabbit and anti-N-WASP polyclonal mouse antibodies) were incubated at room temperature for 1 h. Phalloidin-rhodamine was incubated together with the secondary antibodies conjugated with Alexa 488 and Alexa 647, respectively. Samples were mounted in Fluorosave (Calbiochem) and analyzed with a 60× 1.42 NA objective in an Olympus IX81 Fluoview FV1000 confocal laser scanning microscope (Olympus, Tokyo, Japan). Acquisition settings satisfying the Nyquist sampling criteria were used. Stacks of optically sectioned (0.3 µm in z step) cells were acquired, and the fluorescent signal overlap between N-WASP and *Brucella* was quantified in the relevant voxels.

### Analysis of protein expression and subcellular localization

#### Whole bacteria

*Brucella* whole-cell extracts were resuspended in Laemmli sample buffer and heated to 100°C for 5 min. Samples were submitted to SDS-PAGE (10% or 15% depending on the assay) and transferred to nitrocellulose membranes. The presence of 3×FLAG tagged proteins was carried out by immunoblot analysis using mouse anti-Flag M2 monoclonal antibody (Sigma-Aldrich dilution, 1:5,000) and IRDye secondary anti-mouse antibody (LI-COR, Inc., 1:20000).

#### Total membranes preparation

Total membrane fractions were prepared as previously described (37). Briefly, cells were harvested by centrifugation at 8,000 × *g*, resuspended in buffer A (15 mM Tris-HCl [pH 8], 0.45 mM sucrose, 8 mM EDTA [pH 8], 0.4 mg/mL, lysozyme 5 mg/mL) and incubated for 15 min at 4°C. Then, cells were centrifuged (8,000 × *g*, 4°C, 15 min) and sonicated in buffer B (50 mM Tris-HCl [pH 7.6], 5 mM MgCl2, 2 mM phenylmethylsulfonyl fluoride [PMSF], deoxyribonuclease I [DNase]). The sonicated cells were centrifuged, and supernatant was recovered. Supernatant was diluted 1:4 in buffer C (1 M Tris-HCl [pH 8.0], 1 mM PMSF), and insoluble membrane fractions were recovered by ultracentrifugation (10 ppi, 4°C, 90 min, [Airfuge-Beckman Coulter]) and the pellet was homogenized in 50 mM Tris-HCl, pH 8.0. Pellets and supernatants were stored until western blot analysis.

#### Periplasmic and cytoplasmic localization assay

Fractionation assays were performed as described previously (35). *B. abortus* strains were grown in TSB for 16–24 h at 37°C until an OD_600_ of 1 was reached, and 2.5 × 10^10^ cells were centrifuged for 10 min at 3,300 × *g*. The pellets were washed with PBS, centrifuged for 10 min at 3, 300 × *g*, and resuspended in 1 mL of 0.2 M Tris-HCl (pH 7.6). One milliliter of 0.2 M Tris-HCl (pH 7.6), 1 M sucrose, and 0.25% Zwitterion 3–16 solution was added to the cell suspension and incubated for 10 min at room temperature. The samples were centrifuged for 30 min at 8,000 × *g*, and the pellets were separated from the supernatants and stored at −20°C until used.

### Bioinformatics analysis

#### Disorder and functional site prediction

Signal peptide prediction was performed using SignalP6.0 slow mode. DeepTMHMM was used to predict transmembrane domains.

#### Protein alignment

Homologs for Bab2_0195 (UniProt ID: Q2YJ33) were retrieved from UniProt covering 43 species and 11 genera. Redundant sequences from the same species were removed. Sequences were aligned with Clustal Omega (38), sorted by pairwise distance, and displayed in JalView (39).

## RESULTS

### Identification of Bab2_0195 as a virulence factor that promotes niche formation

**Fig 1:**
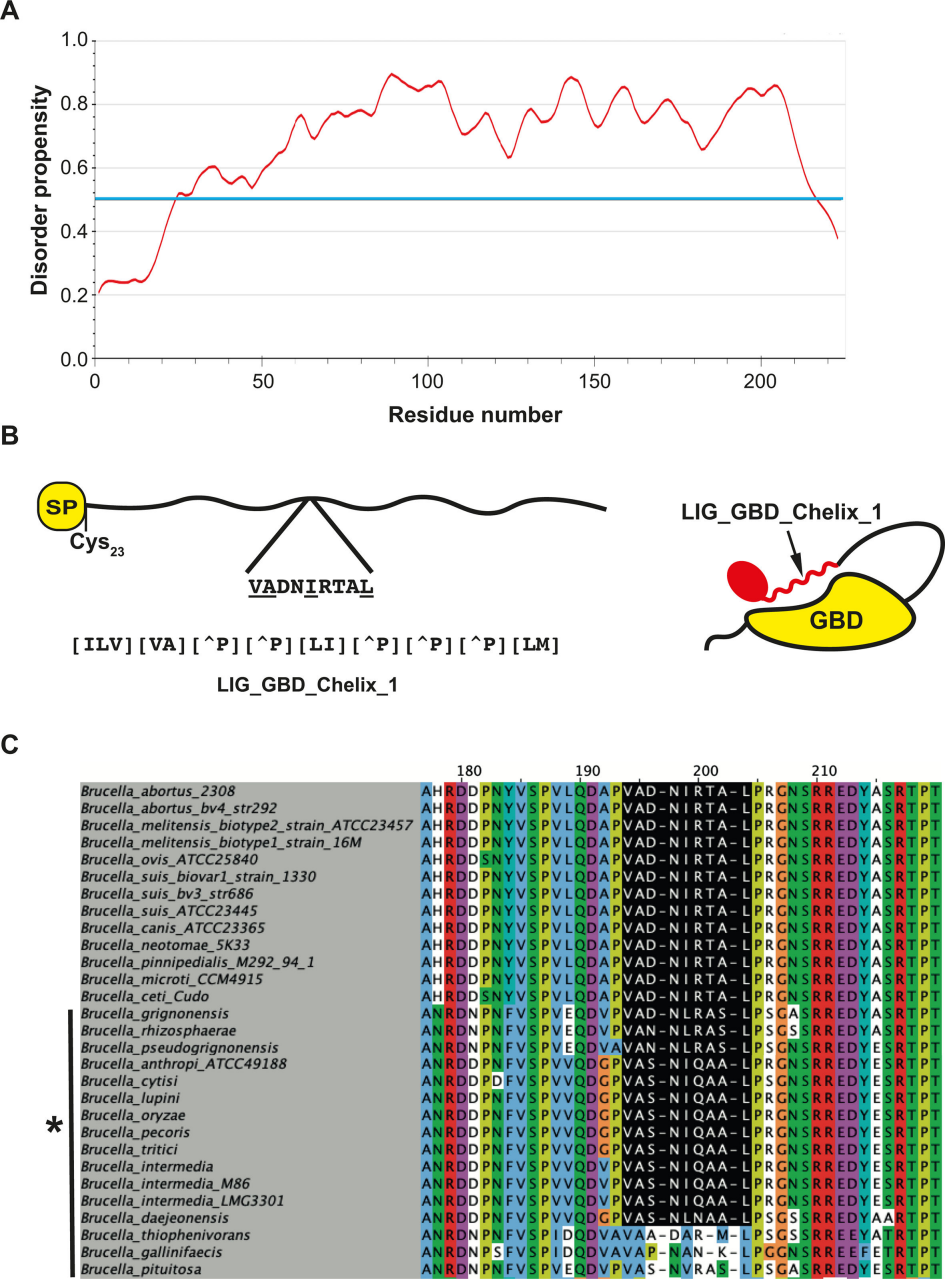
Identification of *bab2_0195* as a candidate effector. (**A**) Disorder propensity plot of Bab2_0195. The blue line indicates the threshold above which a polypeptide region is considered intrinsically disordered. (**B**) Schematic representation of Bab2_0195 showing the signal peptide at the N-terminus of the protein (SP/TM) as well as the predicted lipidated Cysteine 23. The inset shows the region where the actin regulatory ELM:LIG_GBD_Chelix_1 SLiM candidate is present with an amino acid sequence matching the motif regular expression. Below is the regular expression for the SLiM as defined in the ELM entry (41). To the right, a schematic representation of N-WASP in its autoinhibited state with the GBD domain (yellow) interacting with a region (helix in red) that is displaced by the LIG_GBD_Chelix_1 motif present in Bab2_0195. (**C**) Amino acid sequence alignment of the region of Bab2_0195 that harbors the N-WASP-binding SLiM with the *Brucella* species that have the protein. The species marked with an asterisk are former *Ochrobactrum* spp.

**Fig 2:**
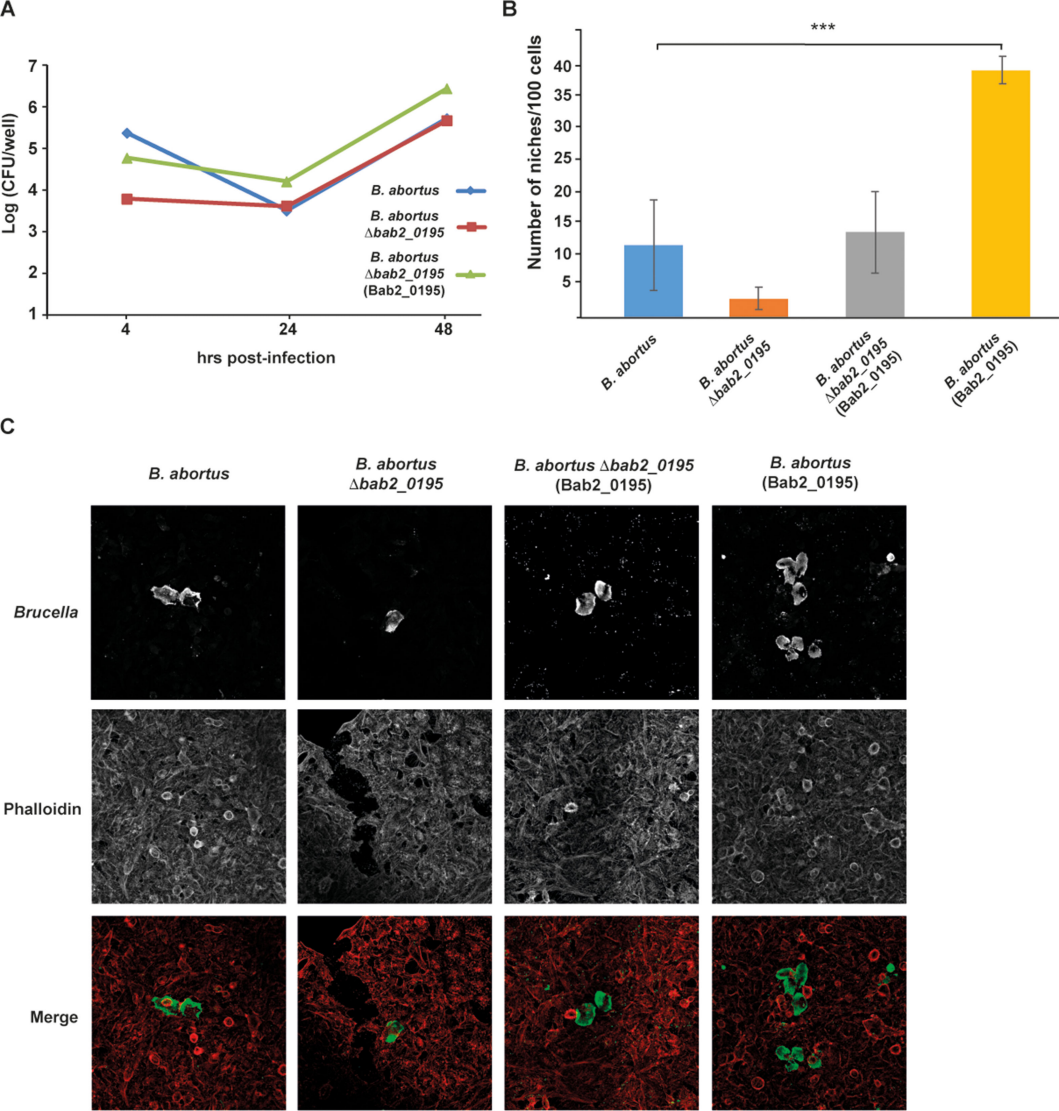
Bab2_0195 codes for a protein that promotes niche formation. (**A**) Intracellular replication in HeLa cells of the *B. abortus* wild type, *B. abortus* Δ*bab2_0195,* and *B. abortus* Δ*bab2_0195* (Bab2_0195) strains. (**B**) Quantification by immunofluorescence microscopy of the number of niches per 100 HeLa cells at 48 h post-infection with the *B. abortus* wild type, *B. abortus* Δ*bab2_0195*, *B. abortus* Δ*bab2_0195* (Bab2_0195), and *B. abortus* (Bab2_0195) strains. The mean and standard deviation for 100 measurements per strain are shown. ***P < 0.001, one-way ANOVA Tukey post-hoc test. (**C**) Representative immunofluorescence microscopy images of the quantification are shown in B. Green, *Brucella*; red, phalloidin. Three hundred cells per experiment were counted.

With the goal of identifying potential proteins of *B. abortus* with actin-modulating activities, we performed an *in-silico* analysis of the complete genome of *B. abortus* 2308 (40) using the Eukaryotic Linear Motif (ELM) resource searching initially for intrinsically disordered regions and, within them, for the presence of putative short-linear motifs (SLiMs) with actin modulatory activities (41). The result of this search revealed several proteins with SLiM candidates related with putative actin modulating properties (Table S1). Out of the candidates identified one was highlighted, *bab2_0195*, a gene present in the genus *Brucella* as well as in other bacteria (almost all of them alphaproteobacteria) and with no homology to other known proteins in the database (Fig. 1C; Fig. S1A). *Bab2_0195* encodes for a protein of 223 amino acids with a predicted N-terminal signal peptide (predicted cleavage position at amino acid 22) followed by a cysteine (Cys_23_) predicted to be lipidated (Fig. 1B). Essentially, the entire Bab2_0195 coding region is predicted to be intrinsically disordered, and therefore, SLiMs present in the sequence should be accessible to interact with their partner proteins (Fig. 1A). We identified a stretch of 9 amino acids (residues 125–133) with a pattern match to the ELM resource entry LIG_GBD_Chelix_1 (ELM motif pattern [ILV][VA][^P] [^P][LI] [^P] [^P] [^P][LM]). We considered it interesting that this candidate actin regulatory motif overlaps with the amphipathic alpha helix that binds the GTPase-binding domain (GBD) of N-WASP (Fig. 1B; Fig. S1B). To determine if this protein plays a role in the virulence of *B. abortus,* we constructed a deletion mutant and performed an intracellular replication assay in HeLa cells as described in Materials and Methods. The *B. abortus* Δ*bab2_0195* strain showed a significant defect in the capacity to survive in HeLa cells that could be detected as early as 4 h post-infection (Fig. 2A). This phenotype was complemented when the gene was expressed in *trans* in the deletion strain. This phenotype was also observed in J774 A.1 cells (Fig. S2). To determine how early the defect in the infection could be detected, we performed antibiotic protection assays to measure the viable intracellular bacteria at 2, 3, and 4 h post-infection. As can be observed in Fig. S3, we detected a defect in the number of intracellular bacteria of the *B. abortus* Δ*bab2_0195* as early as 2 h post-infection. These results indicate that Bab2_0195 plays a role very early after internalization or even during the invasion process. Because the strain carrying the deletion mutant showed a defect during the early phases of the intracellular life cycle, we assessed if this defect affected the ability to establish replicative niches measured by indirect immunofluorescence (see Materials and Methods). For this analysis, we included the following strains: *B. abortus*, *B. abortus* Δ*bab2_0195*, *B. abortus* Δ*bab2_0195* complemented (*B. abortus* [Bab2_0195]), and *B. abortus* overexpressing the Bab2_0195 3×Flag from a multicopy plasmid. The *B. abortus* Δ*bab2_0195* strain showed a significant reduction in its ability to establish intracellular replicating niches at 48 h post-infection compared to the wild-type parental strain (Fig. 2, panels B and C). Interestingly, wild-type *B. abortus* also expressing Bab2_0195–3×Flag showed a ~300% increment in the number of replicative niches compared to the parental strain. This phenotype was also confirmed by antibiotic protection assays that showed that the strain expressing Bab2_0195 from a plasmid was more efficient in replicating intracellularly (Fig. 2A). These results demonstrate that the product of *bab2_0195* is a virulence factor that participates during the early stages of the intracellular cycle and that this activity promotes the establishment of replicative niches. For these reasons, we name *bab2_0195 npeA*, for niche promoting effector A.

### NpeA is a type IV secretion effector with a periplasmic intermediary

**Fig 3:**
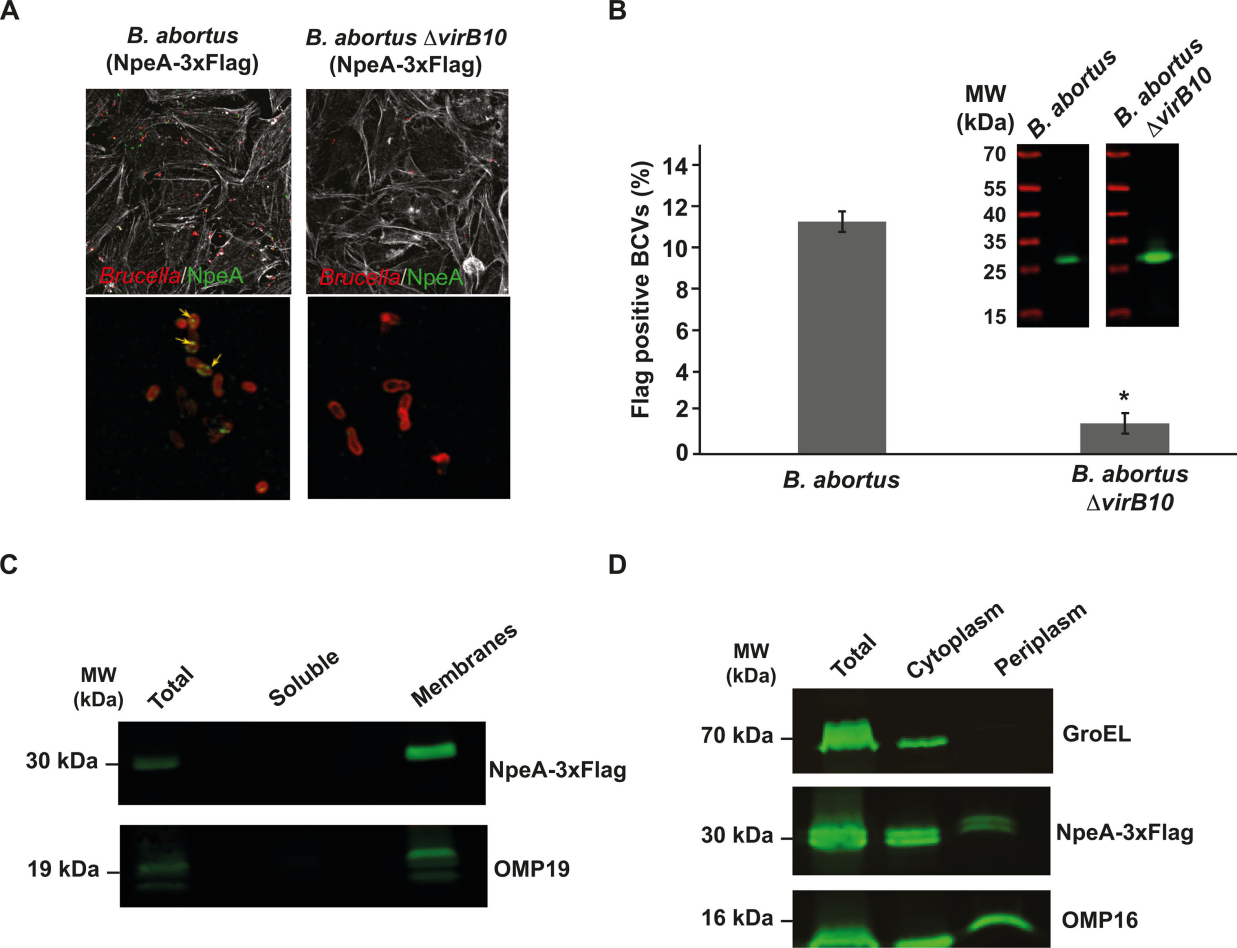
NpeA is a Type IV secretion substrate with a periplasmic intermediary. (**A**) Confocal immunofluorescence microscopy images of HeLa cells infected with the *B. abortus* wild type or *B. abortus* Δ*virB10* strains expressing NpeA-3×Flag. Cells were infected and fixed 2 h post-infection. Red, *Brucella*; green, Flag; white, phalloidin. The lower panels show blow-up images of the NpeA-Flag staining in the *B. abortus* and *B. abortus* Δv*irB10* strains. Arrows indicate the positive staining for Flag. (**B**) Quantification of the secretion of NpeA-3×Flag in the *B. abortus* wild type or *B. abortus* Δ*virB10* strains as determined by the number of Flag-positive BCVs. Inset: western blot showing the expression of NpeA-3×Flag in both strains. (**C**) Western blot analysis of total membranes or soluble fraction (supernatant) prepared with the *B. abortus* (NpeA-3×Flag) strain. NpeA-3×Flag, Flag staining; Omp19, outer membrane protein 19. (**D**) Western-blot analysis of the subcellular localization of NpeA-3×Flag fusion performed with the *B. abortus* (NpeA-3×Flag) strain. NpeA-3×Flag, Flag staining; Omp16, outer membrane protein 16; GroEL, cytoplasmic chaperonin.

The fact that the Δ*npeA* mutant strain showed less intracellular bacteria at very early time points post-infection prompted us to evaluate if the protein encoded by *npeA* is secreted by *B. abortus* and if this secretion is VirB/Type IV dependent. For this, we expressed NpeA tagged with a 3×Flag in the *B. abortus* wild type and also in the *B. abortus* Δ*virB10* deletion mutant strain, infected HeLa cells, and, at 2 h post-infection, determined secretion by immunofluorescence as indicated in Materials and Methods. We observed NpeA signal spots associated with the bacteria very early during infection, and this pattern was completely abolished in the Δ*virB10* background (Fig. 3A and B). Altogether, these results demonstrate that NpeA is a virulence factor that promotes the formation of intracellular replication niches and that it is secreted early after infection in a VirB/Type IV-dependent manner.

NpeA has a predicted canonical signal peptide for periplasmic localization followed by a cysteine predicted to be potentially lipidated. We have described that some *Brucella* Type IV effectors have periplasmic intermediaries (35, 42). To determine if this is also the case for NpeA, we initially performed a membrane fractionation experiment as indicated in Materials and Methods with the NpeA-3×Flag expressing strain. Most NpeA partitioned with the total membrane fraction, which could be due to a mild association with either the inner or the outer membranes (Fig. 3C). To discern between these two possibilities, we performed a periplasmic fractionation to separate protoplasts (inner membranes and cytoplasm) from periplasmic and outer membranes (43). A significant amount of NpeA co-partitioned with the outer membrane protein 16 (OMP16) in the periplasm fraction, which indicated, together with the fact that it fractioned with total membranes that the protein is most probably associated with the outer membrane (Fig. 3D). As an additional control, Fig. S4 shows that in the Δ*virB10* background, NpeA still fractions with membranes indicating that its outer membrane localization is not VirB dependent, but the secretion is.

### NpeA interacts with N-WASP

**Fig 4:**
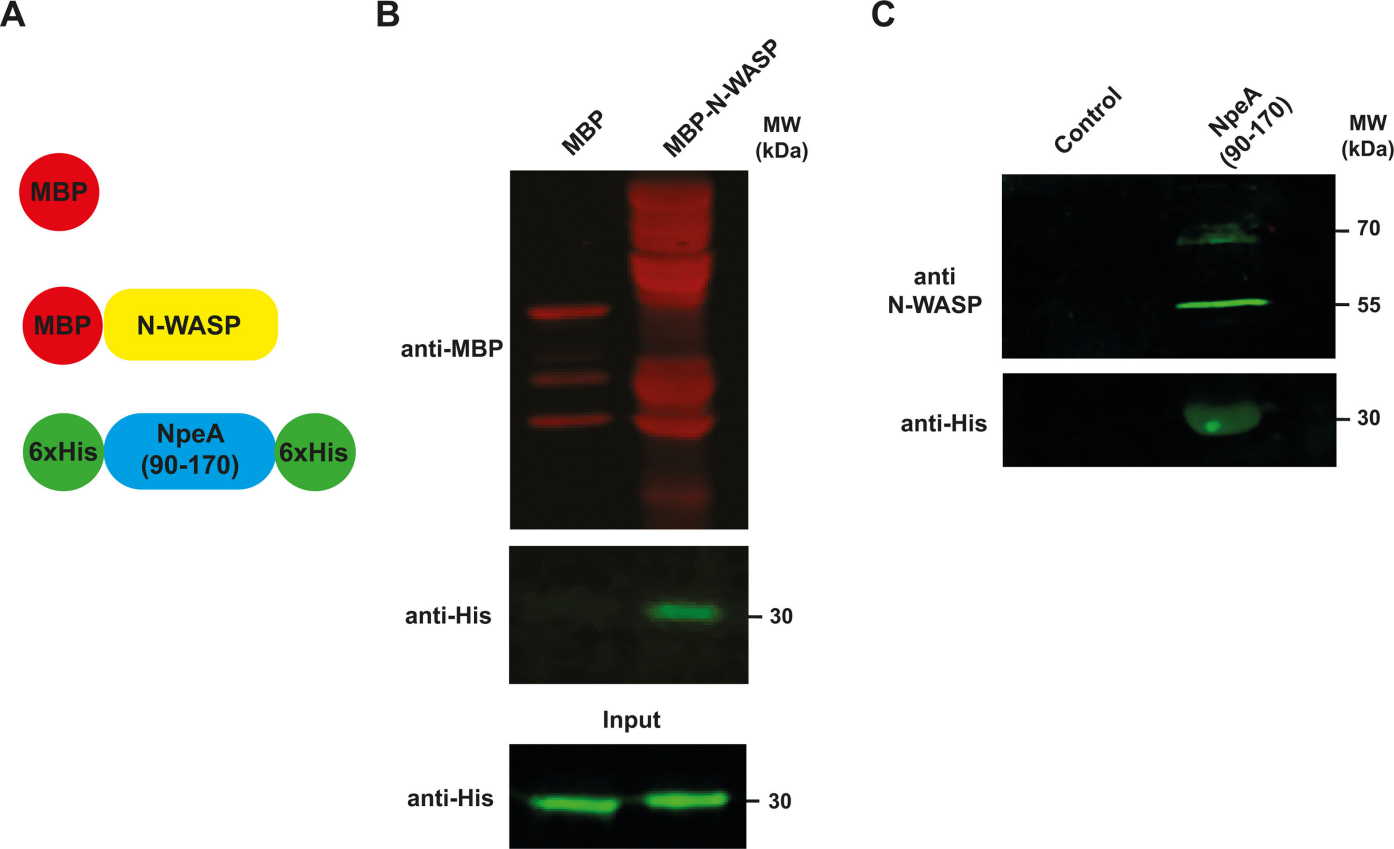
NpeA interacts with N-WASP. (**A**) Schematic representation of the recombinant proteins used in the experiments shown in panels B and C. (**B**) *In vitro* interaction assays. Western blot analysis of a pull-down assay was performed with recombinant NpeA-His and MBP-N-WASP proteins, and the resin-bound fraction was developed with anti-MBP and anti-6×His antibodies. As a negative control MBP alone was used. Input: prior to the pull-down assay, 20 µL of each sample was removed for analysis. (**C**) *Ex vivo* interaction assays. NpeA-His was immobilized on a metal-chelating affinity column, washed, and total cell extracts from HeLa cells injected. After extensive washing, the proteins retained were eluted and analyzed by western Western blot with anti-His and anti-N-WASP antibodies. As a negative control, a column not preloaded with NpeA-His was used.

**Fig 5:**
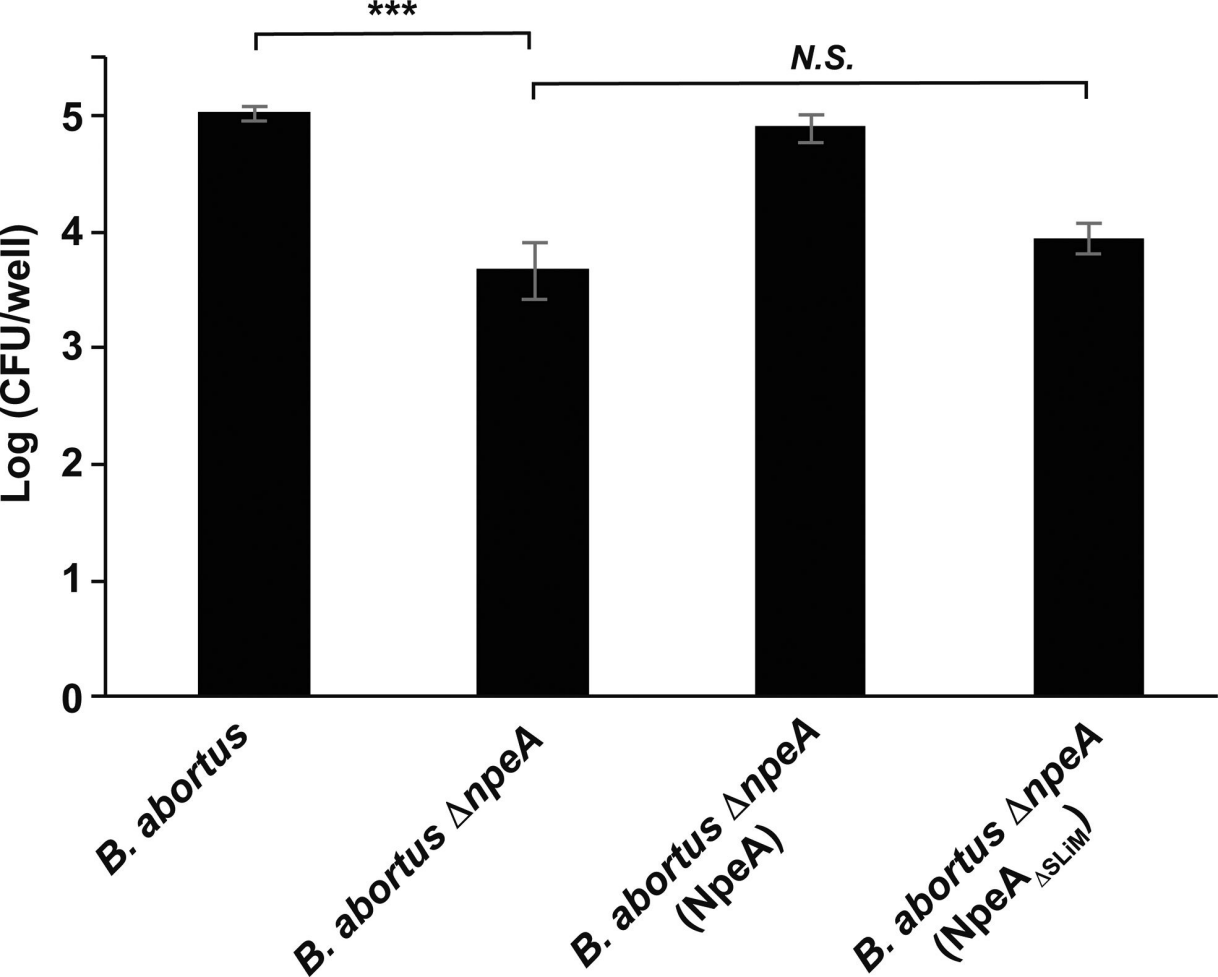
The short linear motif in NpeA is necessary for the intracellular activity of the protein. Intracellular CFUs in HeLa infection assays at 4 h post-infection with the *B. abortus* wild type, *B. abortus* Δ*npeA*, *B. abortus* Δ*npeA* (NpeA), and *B. abortus* Δ*npeA* (NpeA_ΔSLiM_) strains. ***P < 0.001, one-way ANOVA (post-hoc Tukey test).

Thus far, our results indicate that NpeA is a type IV secretion substrate that is involved in the intracellular replication of the bacterium, likely by promoting niche formation. Since the gene product was identified as harboring a putative N-WASP interacting short linear motif, we initially examined the capacity of NpeA to directly interact with N-WASP by two different methodologies: *in vitro* pull-down and interaction *ex vivo*. For the *in vitro* pull-down, we expressed and purified a recombinant soluble fragment of NpeA (encompassing amino acids 90–170 that contains the SLiM, NpeA_90–170_) fused to a double 6×His tag (N- and C-terminal, see Materials and Methods and Fig. 4A), and full-length N-WASP fused to the maltose-binding protein (MBP-N-WASP, Fig. 4A). Pull-down was performed as indicated in Materials and Methods. The resin pre-loaded with the MBP-N-WASP fusion protein was able to pull down NpeA_90–170_, while the one loaded with MBP alone did not (Fig. 4B). To further confirm this interaction, we analyzed the binding of endogenous N-WASP to NpeA in an *ex vivo* interaction assay. The NpeA_90–170_ fused to the 6×His tag was loaded into a Sepharose-nickel column, washed extensively, and afterward, a cell lysate of HeLa cells was injected into the NpeA_90–170_ pre-loaded column (see Materials and Methods). After extensive washings, the retained proteins were eluted, and the eluates were analyzed by Western blot with anti N-WASP and anti-His antibodies. The negative control was a column not loaded with NpeA_90–170_. Only the column loaded with the NpeA_90–170_ protein was capable of retaining endogenous N-WASP from HeLa protein extracts, confirming the specificity of the interaction with NpeA_90–170_ (Fig. 4C). It is important to highlight that, as has been reported in several papers (26, 44, 45), N-WASP is always observed as a double band of 70 and 55 kDa. Altogether, our results demonstrate a direct interaction between NpeA and N-WASP. To determine if the N-WASP-binding SLiM present in NpeA is necessary for its capacity to promote intracellular replication, we complemented the *B. abortus* Δ*npeA* strain with the wild-type NpeA and NpeA_ΔSLiM_ proteins and performed an intracellular replication assay in HeLa cells. As we have shown above, at 4 h post-infection, the *B. abortus* Δ*npeA* strain showed a significant defect in the intracellular survival compared to the wild-type bacterium (Fig. 5). Strikingly, only the wild-type NpeA protein complemented this phenotype, as the strain expressing the NpeA_ΔSLiM_ construct behaved as the deletion mutant.

**Fig 6:**
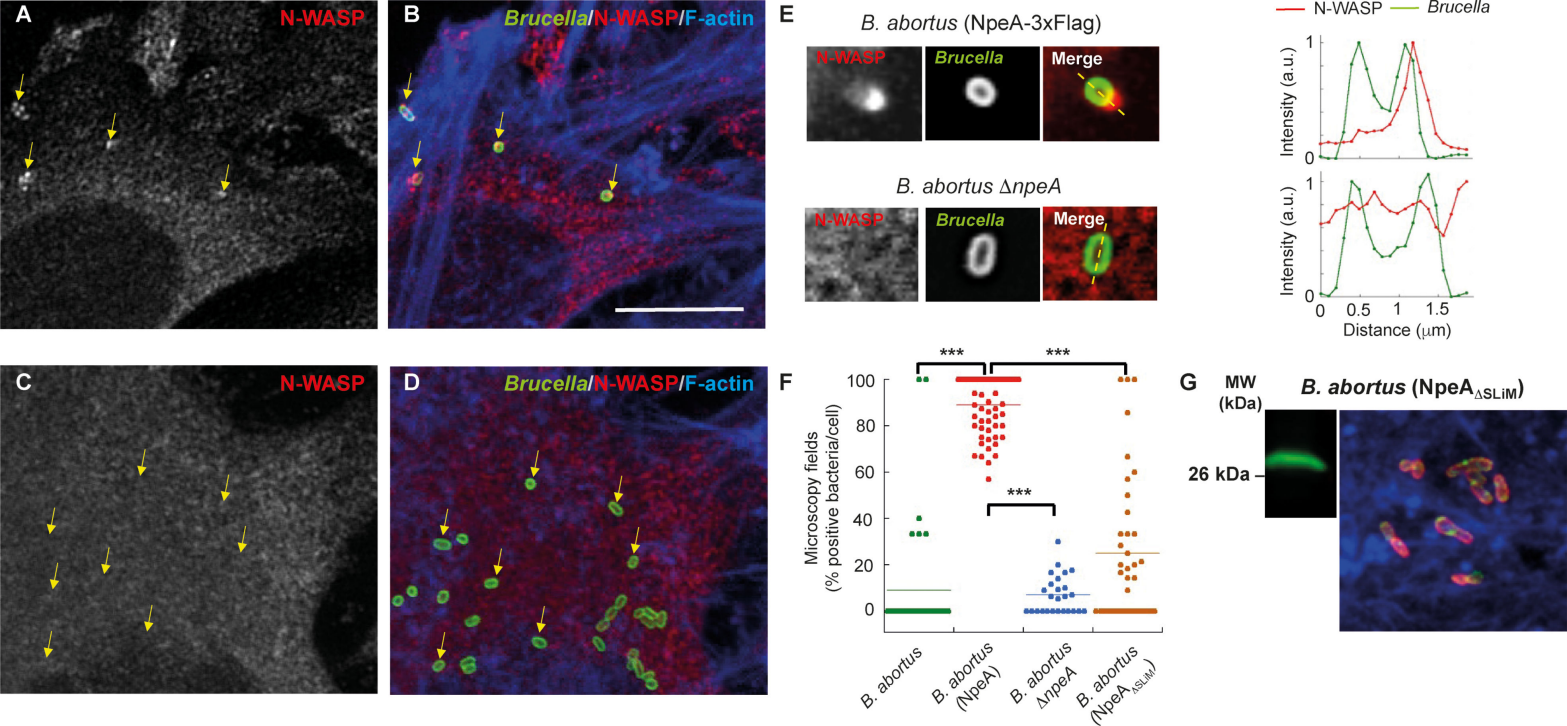
Endogenous N-WASP is recruited to *Brucella* during infection in an NpeA and SLiM-dependent manner. (**A–D**) HeLa cells were infected for 60 min with either *B. abortus* wild type, *B. abortus* Δ*npeA*, *B. abortus* expressing Flag-tagged NpeA [*B. abortus* (NpeA)] or *B. abortus* expressing a NpeA with the short linear motif deleted (ΔSLiM), then fixed and triple stained for *Brucella* (green), N-WASP (red), and F-actin (blue). Fluorescent cells were analyzed by confocal microscopy. Representative panels of *B. abortus* (NpeA) (**A and B**) and *B. abortus* Δ*npeA* (**C and D**) infected cells. Note the presence of N-WASP spots associated with bacteria when NpeA is overexpressed (**A and B**) but not when it is deleted (**C and D**) (yellow arrows). Bar = 10 µm. (**E**) High magnifications of single bacteria, either overexpressing [*B. abortus* (NpeA)] or lacking (*B. abortus* Δ*npeA*) NpeA. Note the tight association of an N-WASP spot with the external side of a bacteria overexpressing NpeA. Plot profiles of each fluorescent signal are shown on the right. The peaks of the green signal correspond to the bacterial cell wall. (**F**) Quantification of overlapping events between N-WASP spots and bacteria. Data are expressed as the percentage of positive bacteria per HeLa cell. The horizontal line represents the mean of each data set. Statistical significance was analyzed using the nonparametric Kruskal-Wallis test followed by a Dunn’s post test (***P = 0.0001). (**G**) Left panel, western blot showing the expression of NpeA_ΔSLiM_ (Flag-tagged) in *B. abortus*. Right panel, secretion of NpeA_ΔSLiM_ (Flag-tagged) in infected HeLa cells at 2 h post-infection as performed in Fig. 3. Red, *Brucella*; green, Flag; blue, phalloidin.

To further corroborate the interaction between N-WASP and NpeA in the cellular context, we infected HeLa cells with the strains *B. abortus*, *B. abortus* expressing Flag-tagged NpeA (NpeA), *B. abortus* Δ*npeA* or *B. abortus* (NpeA_ΔSLiM_) for 1 h and subsequently fixed and processed cells for simultaneous immunofluorescence detection of endogenous N-WASP and *Brucella* (see Materials and Methods). The most dramatic difference was observed between the *B. abortus* (NpeA) and the *B. abortus* Δ*npeA* strains (Fig. 6A through D). In the first case, most bacteria had one, and sometimes more, strong N-WASP spots associated with them (arrows in panels A and B). In contrast, almost none of the *B. abortus* Δ*npeA* bacteria overlapped with N-WASP spots (see arrows in panels C and D). At high magnification, intensity profiles of line scans taken through individual bacteria clearly showed that N-WASP spots tightly associate with the external face of the bacterial cell wall (Fig. 6E). The N-WASP spots were not evident in wild-type *Brucella*, and they were observed with a low frequency in the *B. abortus* (NpeA_ΔSLiM_) (data not shown). Quantification of the N-WASP signal overlapped with individual bacteria confirmed the qualitative observations (Fig. 6F). Taken together, these results indicate that NpeA has the capacity to recruit N-WASP to the *Brucella*-containing vacuole and that, most likely, the GBD ligand peptide is playing a central role in this activity.

## DISCUSSION

*Brucellaceae* spp. are facultative intracellular bacteria that can infect, survive, and multiply in a variety of host cell types, *in vivo* and *in vitro*. Invasion of a susceptible host cell is crucial for bacterial replication and propagation (30). After internalization, the *Brucella*-containing vacuole or BCV progresses through the endocytic pathway acquiring early and late endosomal markers (29). However, even though endosomal BCV acidifies to a pH range of terminal lysosomes and acquires lysosome markers (such as LAMP1), it can defy the lysosomal degradative fate interacting with membranes of the endoplasmic reticulum (ER) and establishing a replicative niche. This subsequent process is dependent on the activity of VirB T4SS effectors (46, 47). After bacteria replication is associated with the ER, replicative BCVs (rBCVs) are remodeled to acquire autophagic features, which eventually fuse with multivesicular bodies and egress from the host cells (48, 49).

To exploit host cell signaling pathways, many bacteria have effector proteins with eukaryotic-like domains that facilitate protein-protein interactions required for host cell subversion. Among these effectors, which have multiple potential targets, regulators of actin cytoskeletal dynamics are often represented (2, 3, 50). Although several steps of the intracellular cycle of *Brucella* are known with relative detail, the molecular interactions of *Brucella* with the host actin cytoskeleton are mostly unknown. A first clue obtained during our bioinformatics search was the identification of a GBD-ligand motif in the *Brucella* protein NpeA, which mimics a conserved motif present in WASP and N-WASP and has been implicated in an auto-inhibitory mechanism of the NPF (12, 16, 51). At least two effector proteins from pathogenic bacteria, the enterohemorrhagic *E. coli* EspFu (23), and *Chlamydia trachomatis* TmeA (26, 44) have been found to contain a similar motif to subvert N-WASP regulation. In the case of *E. coli* EHEC, delivery of EspFu into the host cell results in WASP recruitment to the plasma membrane and the formation of pedestals where bacteria adhere and persist (22). *C. trachomatis* orients its Type III secretion system to the contact site with the host cell membrane (52) and actin-rich macropinosomes, phagocytic cups, and filopodia are frequently observed to be surrounding the adhered bacterium (26, 53). *C. trachomatis* transiently colocalizes with N-WASP at the plasma membrane, an event that is abolished in the absence of TmeA. A third effector, IcsA from *Shigella flexneri*, shares with EspFu and TmeA the ability to bind to N-WASP and promote Arp2/3-dependent actin polymerization (25, 54). However, the search of the ligand GBD C-helix motif within the IcsA sequence did not retrieve any hit. Likely, the recognition of N-WASP by IcsA may differ from EspFu, TmeA, and NpeA. Indeed, while EspFu and TmeA are capable of binding to the VCA-binding site of the GBD domain of both N-WASP and the related WASP protein, IcsA only binds to N-WASP, a selectivity that could be attributed to the dual recognition of N-WASP at the VCA-binding site of GBD and the N-terminal WH1 domain (23, 54). Despite the structural details, all these factors appear to activate N-WASP by competing directly for the VCA-binding site of the GBD domain, breaking the autoinhibitory mechanism that restricts the access of Arp2/3 to the VCA domain.

In the present work, we show that NpeA binds directly to N-WASP. The GBD domain of N-WASP (residues 195–274) is a large region composed of the Cdc42/Rac-interactive binding (CRIB, residues 195–253) motif, followed by a β-hairpin and an α helix (layer 1) as well as a VCA binding site that ensures the autoinhibited state (layer 2 [16]). Like TmeA, NpeA contains a single copy of the GBD ligand motif, whereas EspFu harbors five to seven copies. While a single motif in TmeA is sufficient to promote N-WASP activation (44), the motif repeats present in EspFu have been shown to enhance activation (55). Although we did not map the minimal binding region in N-WASP , the similarity between the ligand GBD C-helix motif of NpeA and that of EspFu and TmeA suggests that NpeA may bind to the VCA-binding site of the GBD domain. In support of this possibility, a mutant of NpeA with a deleted GBD binding motif, NpeA_ΔSLiM_, was unable to complement the replication defect of the *B. abortus* Δ*npeA* strain and significantly reduced the N-WASP signal associated with the NpeA-3×Flag strain. Of note, at 60 min post-infection, bacteria of the NpeA-3×Flag expressing strain frequently were surrounded by filopodia-like projections from the dorsal cell membrane (not shown), a feature that was never seen in the Δ*npeA* strain. It is possible that NpeA plays a role analogous to TmeA related to the capture of dorsal filopodia (26, 53), and that at the molecular level, NpeA functions like TmeA, EspFu, and IcsA, by binding to the VCA binding site of the GBD domain of N-WASP and unleashing the VCA region for downstream interactions with the Arp2/3 complex. Future studies should confirm these possibilities.

The participation of the actin cytoskeleton in *Brucella* internalization was demonstrated using actin-depolymerizing drugs such as cytochalasin D although the degree of actin accumulation at the site of entry is moderate compared to other pathogens such as *Salmonella* (56, 57). A transient peak of Cdc42 activation also accompanies the *Brucella* uptake (57, 58). To our knowledge, no activator of Cdc42 has been reported for *Brucella*. The present study is the first to identify a Type IV-dependent effector which promotes the *Brucella* intracellular life cycle through the manipulation of N-WASP. Several lines of evidence including (i) the lack of N-WASP recruitment to the NpeA null mutant, (ii) complementation of NpeA with a NpeA variant that is unable to bind N-WASP does not rescue its defect, (iii) the direct interaction of N-WASP with NpeA, (iv) the association of N-WASP with *Brucella* early after infection, and (v) the dependence of the N-WASP/NpeA association on the NpeA GBD C-helix motif collectively suggest a role of NpeA in the early steps of the intracellular life cycle or the invasion process through the manipulation of N-WASP.

## SUPPLEMENTAL MATERIAL

Figure S1 mbio.00726-24-s0001.tiff Amino acid sequence and ribbon diagram.

Figure S2 mbio.00726-24-s0002.eps Intracellular replication of *B. abortus* strains.

Figure S3 mbio.00726-24-s0003.tif Intracellular survival of *B. abortus* strains.

Figure S4 mbio.00726-24-s0004.tif Western blot analysis.

Table S1 mbio.00726-24-s0005.docx Actin-modulating SLiMs found in the *B. abortus* genome.

ASM does not own the copyrights to Supplemental Material that may be linked to, or accessed through, an article. The authors have granted ASM a non-exclusive, world-wide license to publish the Supplemental Material files. Please contact the corresponding author directly for reuse.
