# Supplementary figures and images for: Brucella NpeA is a secreted Type IV effector containing an N-WASP-binding short linear motif that promotes niche formation
Source: mBio. 2024 Jun 7;15(7):e00726-24. doi: 10.1128/mbio.00726-24 (PMC11253601; doi:10.1128/mbio.00726-24)

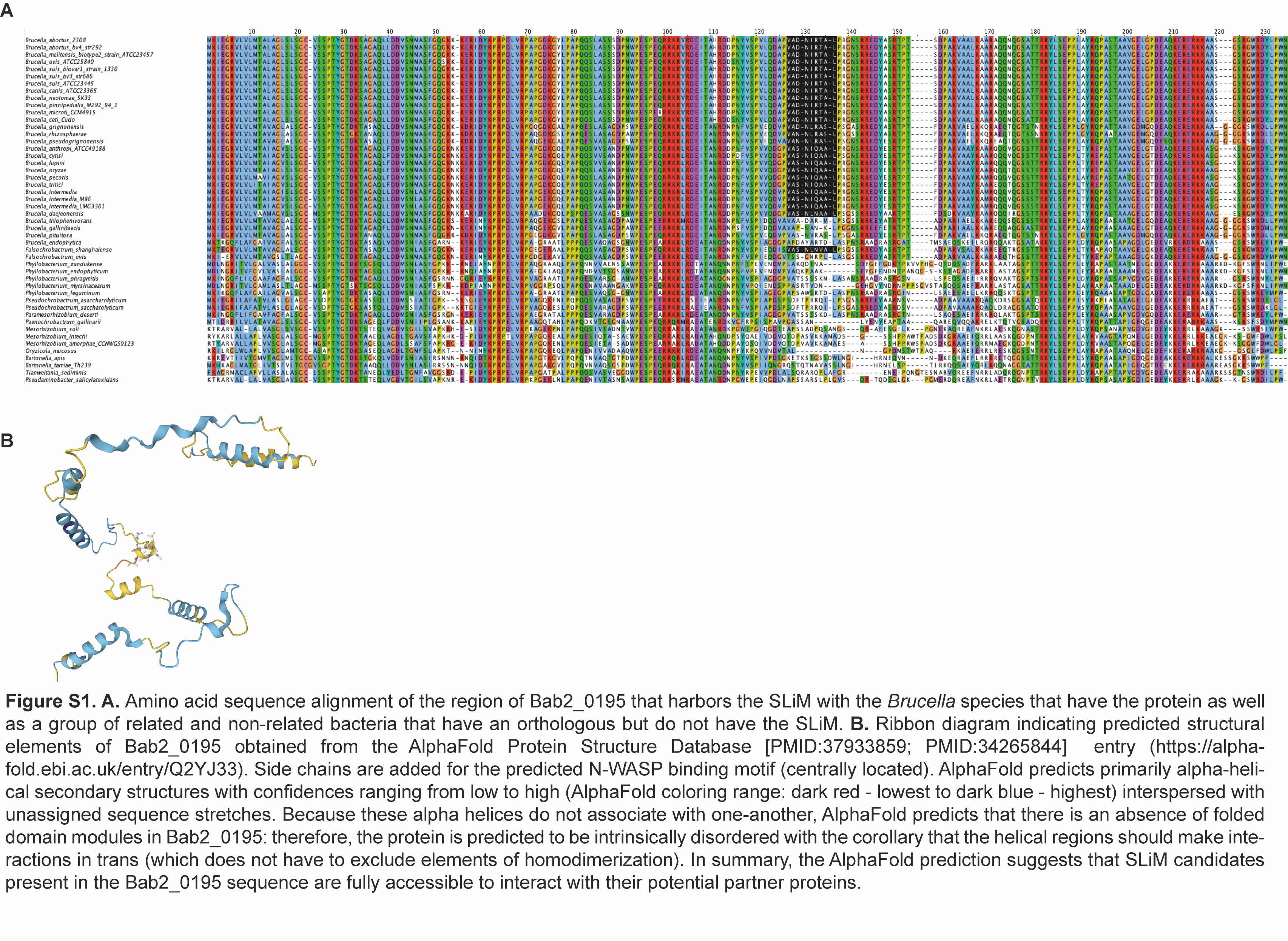

Supplement: Figure S1 — Amino acid sequence and ribbon diagram. [file mbio.00726-24-s0001.tiff]

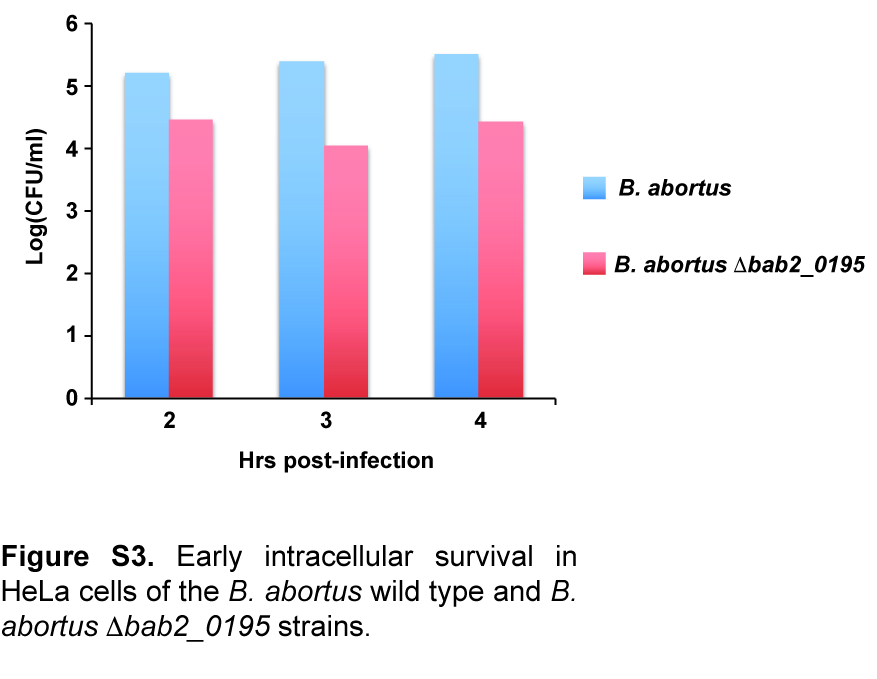

Supplement: Figure S3 — Intracellular survival of B. abortus strains. [file mbio.00726-24-s0003.tif]

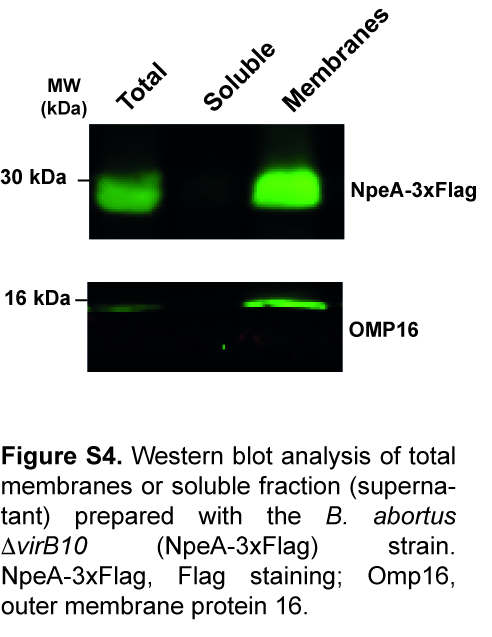

Supplement: Figure S4 — Western blot analysis. [file mbio.00726-24-s0004.tif]
