# Supplementary material for: Brucella NpeA is a secreted Type IV effector containing an N-WASP-binding short linear motif that promotes niche formation
Source: mBio. 2024 Jun 7;15(7):e00726-24. doi: 10.1128/mbio.00726-24 (PMC11253601; doi:10.1128/mbio.00726-24)
Supplement: Table S1 — Actin-modulating SLiMs found in the B. abortus genome. [file mbio.00726-24-s0005.docx]

| **Partner** | **Motif** | **Count** |
| --- | --- | --- |
| Actin | LIG_Actin_WH2_x | 53 |
| WASP | LIG_GBD_Chelix_1 | 4 |
| Vinculin | LIG_Vh1_VBS | 0 |
| EVH1 | LIG_EVH1_1 | 14 |
| Paxillin | LIG_FAT_LD | 0 |
| Profilin | Cand_LIG_PROFILIN_1 | 0 |

**Table S1.** Actin modulating SLiMs found in the B. abortus genome.
